# Functional identification of *PsMYB57* involved in anthocyanin regulation of tree peony

**DOI:** 10.1186/s12863-020-00930-7

**Published:** 2020-11-16

**Authors:** Yanzhao Zhang, Shuzhen Xu, Yanwei Cheng, Jing Wang, Xiangxiang Wang, Runxiao Liu, Jianming Han

**Affiliations:** Life Science Department, Luoyang Normal University, Luoyang, 471022 China

**Keywords:** Transcriptome, *MYB* gene family, Tree peony, *PsMYB57*, Anthocyanin

## Abstract

**Background:**

*R2R3 myeloblastosis* (*MYB*) genes are widely distributed in plants and comprise one of the largest transcription factor gene families. They play important roles in the regulatory networks controlling development, metabolism, and stress responses. Researches on functional genes in tree peony are still in its infancy. To date, few *MYB* genes have thus far been reported.

**Results:**

In this study, we constructed a comprehensive reference gene set by transcriptome sequencing to obtain *R2R3 MYB* genes. The transcriptomes of eight different tissues were sequenced, and 92,837 unigenes were obtained with an N50 of 1662 nt. A total of 48,435 unigenes (77.98%) were functionally annotated in public databases. Based on the assembly, we identified 57 *R2R3 MYB* genes containing full-length open reading frames, which clustered into 35 clades by phylogenetic analysis. *PsMYB57* clustered with anthocyanin regulation genes in *Arabidopsis* and was mainly transcribed in the buds and young leaves. The overexpression of *PsMYB57* induced anthocyanin accumulation in tobacco, and four detected anthocyanin structural genes, including *NtCHS*, *NtF3’H*, *NtDFR,* and *NtANS,* were upregulated. The two endogenous *bHLH* genes *NtAn1a* and *NtAn1b* were also upregulated and may work in combination with *PsMYB57* in regulating anthocyanin structural genes.

**Conclusions:**

Our study offers a useful reference to the selection of candidate *MYB* genes for further functional studies in tree peony. Function analysis of *PsMYB57* is helpful to understand the color accumulation in vegetative organs of tree peony. *PsMYB57* is also a promising resource to improve plant color in molecular breeding.

**Supplementary Information:**

The online version contains supplementary material available at 10.1186/s12863-020-00930-7.

## Background

Myeloblastosis (MYB) transcription factors (TFs) are widely distributed in all eukaryotes and are one of the largest families of TFs in plants. MYB TFs are characterized by a conserved DNA binding domain (DBD) known as the MYB domain near the N-terminus. The MYB domain is comprised of up to four incomplete repeats, each of which consists of about 50–53 amino acids and forms three α-helices [1, 2]. The second and third helices form a helix-turn-helix (HTH) structure, and the third α-helix of each repeat is a DNA-recognition helix that binds specific DNA sequences in the major groove [3, 4]. Typically, the MYB repeat contains three regularly spaced tryptophans, which stabilize the structure of the MYB domain by forming a hydrophobic core [5]. In plants, the first Trp of the third repeat is always substituted by phenylalanine (Phe) or isoleucine (Ile) [3, 6, 7]. Compared with the conservative MYB domain, the C-terminal regions of MYB proteins are more flexible. They typically function as transacting domains (TADs) and are responsible for the regulatory activity of the protein [8].

Based on the repeat number in the DBD domain, MYB proteins are classified into four subfamilies, including R2R3-MYB, R1R2R3-MYB, 4R-MYB, and MYB-related. R2R3-MYB proteins contain two repeats in the DBD domain and constitute the largest group of the MYB family. Currently, 8746 R2R3 MYB sequences are available in the plant transcription factor database (http://planttfdb.cbi.edu.cn/). In some plants with available complete genome sequences, the *R2R3 MYB* family gene has been systematically studied. 126 *R2R3 MYBs* were reported in *Arabidopsis* [9], 123 in *Jatropha curcas* [10], 94 in pineapple [11], and 128 in peach [12]. Since the first plant *MYB* gene *C1* was cloned from maize, functional characterization of the *R2R3 MYB* gene has been conducted in various plants. A summary of *MYB* gene function in *Arabidopsis* showed that these genes play important roles in multiple plant-specific processes, such as primary and secondary metabolism, cell fate and identity, developmental processes, and responses to biotic and abiotic stress [9].

The anthocyanin pathway is a branch of the flavonoid pathway and includes two clusters of coregulated structural genes: early biosynthetic genes (EBG) and late biosynthetic genes (LBG). In the R2R3 MYB family, a small group of members are key factors in regulating anthocyanin biosynthesis and usually interact with bHLH factors to form complexes that regulate structural genes by binding to their promoters. In *Arabidopsis*, three *MYBs*, including *MYB11*, *MYB12*, and *MYB111*, mainly regulate EBGs and control the biosynthesis of flavonols. Four *MYBs*, including *MYB75*, *MYB90*, *MYB113*, and *MYB114*, mainly regulate LBGs. In nature, some tissue colors in plants are caused by the mutation of *MYB* genes. For example, a mutation in the promoters of *Pr* in purple cauliflower [13], *MdMYB10* in red flesh apple [14], and *Ruby* in blood oranges [15] leads to the heterotopic accumulation of anthocyanins. Recently, the overexpression of members in non-model plants have been reported to lead to high levels of anthocyanin accumulation, such as *PtrMYB119* in *Populus trichocarpa* [16], *DcMYB6* in purple carrot [17], *MaAN2* in grape hyacinth [18], and *OjMYB1* in *Oenanthe javanica* [19].

Tree peony are among the most valued ornamental flower with a variety of colors in China. Researches on functional genes in tree peony are still in its infancy. To date, few studies in the *MYB* gene family have been reported. Due to the unpublished and highly complex genome of tree peony, transcriptome sequencing has become a suitable method for obtaining gene sequences. Several transcriptomic studies of tree peony have been made [20–22]. However, such studies have not provided a global genetic overview. In this study, we performed global transcriptome sequencing and constructed a reference gene sequence library of tree peony. The *R2R3 MYB* genes were screened and systematically analyzed in order to provide a foundation for further functional gene research. The anthocyanin regulatory *MYB* gene was functionally characterized, which will facilitate improvements in plant color in tree peony.

## Methods

### Tissue collection and total RNA extraction

*Paeonia suffruticosa* Andr. cv. Er Qiao is a traditional variety in China. Erqiao plants were kindly identified and provided by Luoyang Research Institute of Peony (Luoyang, China), and were grown under field conditions. From five healthy plants, we collected samples of bud, young leaf, red petal, pink petal, petal spot, stamen, pistil, and seed. The samples of each tissue type were pooled. The samples were rapidly frozen in liquid nitrogen and stored at − 80 °C. Total RNA extraction was performed using the CTAB-LiCl method [23], and the contamination of genomic DNA was eliminated using DNase I (TaKaRa, Dalian, China).

### Sequencing and de novo assembly

The cDNA libraries were constructed and sequenced at BGI Co., Ltd. (Shenzhen, China), and sequencing was performed on a BGISEQ-500 platform. The raw sequence data reported in this paper have been deposited in the Genome Sequence Archive [24] in BIG Data Center [25] Beijing Institute of Genomics (BIG), Chinese Academy of Sciences, under accession numbers CRA001327 that are publicly accessible at http://bigd.big.ac.cn/gsa. The raw reads were filtered by removing the adapter sequences, low quality reads, and reads with more than 20% Q < 20 bases. The remaining clean reads generated from each sample were de novo assembled using Trinity [26], and all assembled datasets were further clustered using TGICL to generate a Nr unigene set [27]. The ORFs of all unigenes were predicted using Transdecoder v3.0.1.

### Gene annotation

To predict the functions of the genes, the assembled unigenes were used to query seven public databases using BLAST with an E-value of <1e^− 5^, including the Nr, nucleotide (Nt), SWISS-PROT, InterPro, Gene Ontology (GO), Kyoto Encyclopedia of Genes and Genomes (KEGG), and eukaryotic orthologous group (KOG). Based on the Nr annotation, Blast2GO was used to obtain GO annotations [28], and WEGO software was used to perform GO functional classification for all unigenes [29]. Pathway assignments were also carried out based on the KEGG database.

### Identification of *R2R3-MYB* genes in tree peony

To identify the maximum number of MYB domain-containing sequences, de novo assembled unigenes were used to construct a local database using the BLAST program, and the HMM profile of the MYB DNA-binding domain (PF00249) downloaded from Pfam was used to isolate all possible homologs in tree peony with HMMER 3.0 [30]. The default parameters were adopted, and the cutoff value was set to 0.001. The redundant proteins were removed and sequences without complete ORFs were discarded. The remaining sequences were detected using PROSITE (https://prosite.expasy.org/scanprosite/) and the SMART online service (http://smart.embl-heidelberg.de/) to eliminate any sequences that do not contain the R2R3 MYB domain. Calculation of protein isoelectric point and molecular weight was performed using the ExPASy proteomics server (http://www.expasy.ch/tools/protparam.html). Subcellular localization of proteins was predicted using the online service of Cell-PLoc 2.0 (http://www.csbio.sjtu.edu.cn/bioinf/Cell-PLoc-2/).

### Gene sequence analysis

For conservation analysis of the R2R3 MYB domain in tree peony, all obtained R2R3 MYB proteins were aligned using ClustalW, and the sequence logos for the R2 and R3 repeats were generated using the Weblogo v3 online tool [31]. To examine the phylogenetic relationships and evolutionary history of the *MYB* gene family, full-length proteins of the MYB protein from tree peony and *Arabidopsis* were used to generate a phylogenetic tree based on the neighbor-joining method using MEGA5.0 software [32], and tree nodes were evaluated with 1000 bootstrap replicates. The phylogenetic tree was further modified using the iTOL online service (http://itol.embl.de/itol.cgi). Sequences and functional annotations of all *Arabidopsis* MYB proteins were obtained from the *Arabidopsis* Information Resource (TAIR) database.

### Expression analysis by qRT-PCR

Total RNA was extracted as described above, and the first-strand cDNA was reversed-transcribed with 1 μg of total RNA using the PrimeScript™ 1st Strand cDNA Synthesis Kit (TaKaRa, Dalian, China). qRT-PCR experiments were performed using SYBR® Premix Ex Taq™ II kit (TaKaRa, Dalian, China) on an ABI7500 system according to the manufacturer’s instructions. The thermal cycling conditions were as follows: an initial heat denaturing step at 95 °C for 3 min; followed by 40 cycles of 95 °C for 20 s, 58 °C for 20 s, and 72 °C for 20 s. Each sample was amplified with three biological replicates and three technical replicates. Gene transcription levels were calculated using the 2^−∆∆CT^ comparative cycle threshold method [33]. *Ubiquitin* and *tubulinA1* were used as an internal control for *Paeonia suffruticosa* [34] and tobacco [18], respectively, to normalize the relative expression levels of the analyzed genes. Primers used for PCR amplification are shown in Table S1.

### Plasmid construction and tobacco transformation

To verified assembly genes, ten paired primers were designed to amplifacte randomly selected *MYB* genes (Table S2). Each of the purified PCR products was ligated to pMD19-T vector and transferred into *E.coil* DH5α competent cells. Three clones of each gene were sequenced using sanger sequencing method. The coding region sequence of *PsMYB57* was amplified by PCR using the following primers: forward primer: 5′-ACGCGTCGACATGGAGGGAATGTTAGGATTGAGAAAAG-3′, reverse primer: 5′-ATTGGCTGCAGTTAATTCACCGCTTGCCCTTCAGCACTTAATAG-3′. Following agarose gel electrophoresis, the PCR product was digested with the *Sal*I and *Pst*I enzymes. The target fragment was linked to the pCAMBIA1300–35 s vector. The recombinant plasmid was transferred into *Agrobacterium* strain Gv3101 by electrical transformation. Tobacco plants (*Nicotiana tabacum* L. cv. SR1) were transformed with *Agrobacterium* using a previously described protocol [35], and transgenic lines showing color changes in the leaves were used for further analysis.

## Results

### Transcriptome sequencing and assembly

As the genomic information was very limited for tree peony, we performed a global transcriptome sequencing to identify *MYB* gene family members. Eight libraries were constructed from the total RNA of the tissues, including the bud, young leaf, pink petal, red petal, petal spot, pistil, stamen, and seed. After filtering, each library generated over 6.5 Gb data with Q20 scores over 96.5%. A total of 52.82 Gb data were obtained (Table S3). The data were de novo assembled using Trinity. After further assembly with TGICL, a total of 92,837 unigenes were generated with an N50 of 1662 nt. Among the assembled unigenes, 49,376 were longer than 500 bp, 10,543 were longer than 1000 bp, and 1600 were longer than 1500 bp.

### Functional annotation

Gene functions were predicted by querying seven public databases, and a total of 48,435 unigenes (77.98%) were functionally annotated (Table S4). Among them, 45,046 unigenes (72.52%) obtained hits in the non-redundant (Nr) database, 31,786 obtained hits in the SWISS-PROT database, and 33,713 unigenes obtained hits in the InterPro database. Based on the Nr annotation, the top sequence matches obtained from BLASTX are shown in Fig. S1. The sequences were most similar to *Vitis vinifera* (35.2%), followed by *Nelumbo nucifera* (6.14%), *Theobroma cacao* (6.12%), *Jatropha curcas* (3.99%), and *Prunus mume* (3.39%).

### Identification of *R2R3 MYB* genes in tree peony

To identify *R2R3 MYB* genes in tree peony, the MYB DNA-binding domain (PF00249) was queried in the de novo assembly using HMMER 3.0, and hits with full-length open reading frames (ORFs) were selected. The redundant sequences were removed and the MYB domain of the remaining sequences was further verified using SMART. A total of 57 *R2R3 MYB* genes were ultimately identified. As two *R2R3 MYB* genes in our transcriptome have previously been deposited in Genbank as *PsMYB1* and *PsMYB2*, the remaining 55 genes were provisionally named *PsMYB3* to *PsMYB57* (Genbank accession numbers: MK377190- MK377244). To evaluate the assembly genes, ten randomly selected *MYB* genes were cloned and sequenced using sanger sequencing method. Results showed that the *MYB* genes had 97 to 100% identity with assembly genes. In general, the sanger sequencing results were in rough accordance with the electronic data of assembly. The MYB proteins ranged from 170 (PsMYB33) to 560 (PsMYB9) amino acids in length (Table S5). The predicted isoelectric point of the R2R3-MYB proteins ranged from 4.86 (PsMYB43) to 10.22 (PsMYB3). Subcellular localization analysis revealed that all 57 MYB proteins were localized in the nucleus.

**Fig. 1:**
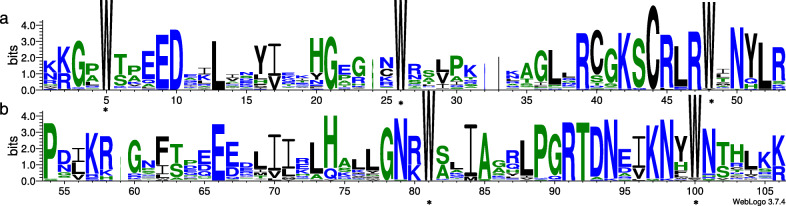
The R2 and R3 MYB repeats are highly conserved across all R2R3 MYB proteins in tree peony. The sequence logos of the R2 (**a**) and R3 (**b**) MYB repeats are based on full-length alignments of all R2R3 MYB proteins. The bit score exhibits the information content for each position in the sequence. Asterisks indicate the conserved tryptophan residues (Trp) in the MYB domain

To investigate the MYB domain features and the conservation of amino acids, sequence logos were generated using the aligned R2 and R3 motifs (Fig. 1). The results indicated an even distribution of a series of highly conserved tryptophan residues (W), which are considered as a hallmark of the MYB domain. In the R2 domain, three highly-conserved W were present at positions 5, 26, and 48, but tyrosine (Y) replaced the W at position 48 in PsMYB38. While two highly conserved W exist at positions 81 and 100 in the R3 domain, the first W residue was generally replaced by phenylalanine (F), isoleucine (I), and leucine (L), and substitutions with the amino acid methionine (M) were also observed in PsMYB52. Furthermore, F and Y substituted the W at the W-100 position in PsMYB17 and PsMYB18, respectively. In addition to the highly conserved W residues, D-10, L-13, G-21, C-44, and R-47 in the R2 repeat, and E-66, G-78, R-91, and T-92 in the R3 repeat were completely conserved. These results reveal that R2R3-MYB proteins are highly conserved in tree peony.

### Phylogenetic analysis of the MYB proteins

**Fig. 2:**
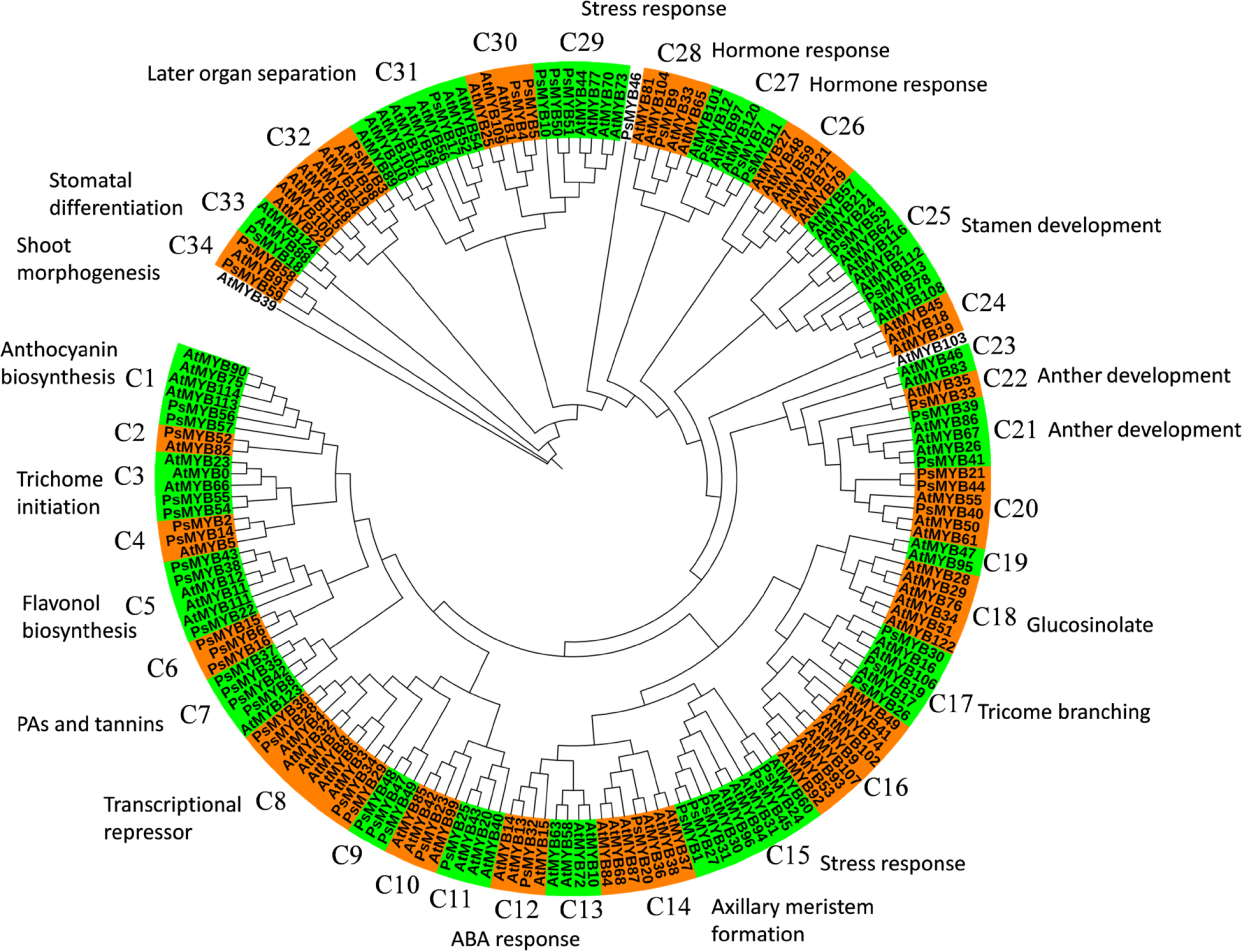
Phylogenetic analysis of R2R3 MYB family genes in tree peony and *Arabidopsis.* The full-length amino acid sequences of MYB proteins were aligned using CLUSTALW and the phylogenetic tree was constructed using the neighbor-joining method in MEGA5.0. Subgroup names are included next to each clade together with a short name, and the putative functions of MYB proteins in tree peony are annotated alongside the names. Green and orange shading are used to distinguish the different clades

To explore the putative function of MYBs in tree peony, a phylogenetic tree was constructed using full-length R2R3 MYBs, including 57 proteins from tree peony and 124 proteins from *Arabidopsis*. As is shown in Fig. 2, a total of 181 MYBs were clustered into 35 clades, whereas the proteins PsMYB46 and AtMYB139 were not grouped into any clade. In 26 of the 35 clades, members from both tree peony and *Arabidopsis* were present. However, in some clades, MYBs were unequally represented. For example, C25, C32, and C33 included just one or two PsMYBs but at least seven AtMYBs, while PsMYB members were more abundant than that in *Arabidopsis* in the C2 and C4 clades. We also detected some species-specific proteins. There were no PsMYB grouped in 7 clade, including clade 9, 10, 14, 21, 22, 26 and 27, whereas clade C7 contained no members from *Arabidopsis*. Overall, our classification of the MYBs corroborates that of *Arabidopsis*.

According to the gene annotation summary in *Arabidopsis* [9], the functions of PsMYBs in different clades were predicted and were found to be involved in a broad range of physiological functions. The functions of the three clades were associated with the regulation of secondary metabolism, including anthocyanin biosynthesis (C1), proanthocyanidins (PAs) and tannis (C4), and flavonol biosynthesis (C5); three clades were associated with determining cell fate and identity, including trichome initiation (C3), trichome branching (C13), and stomatal differentiation (C35); five clades were associated with plant developmental processes, including axillary meristem formation (C23), anther development (C24), stamen development (C25), later organ separation (C32), and shoot morphogenesis (C34); and five clades were associated with responses to biotic and abiotic stress, including abscisic acid response (C15), hormone response (C28, C29), and stress response (C12, C30).

### *R2R3 MYB* genes involved in anthocyanin biosynthesis and their expression profiles in different tissues

**Fig. 3:**
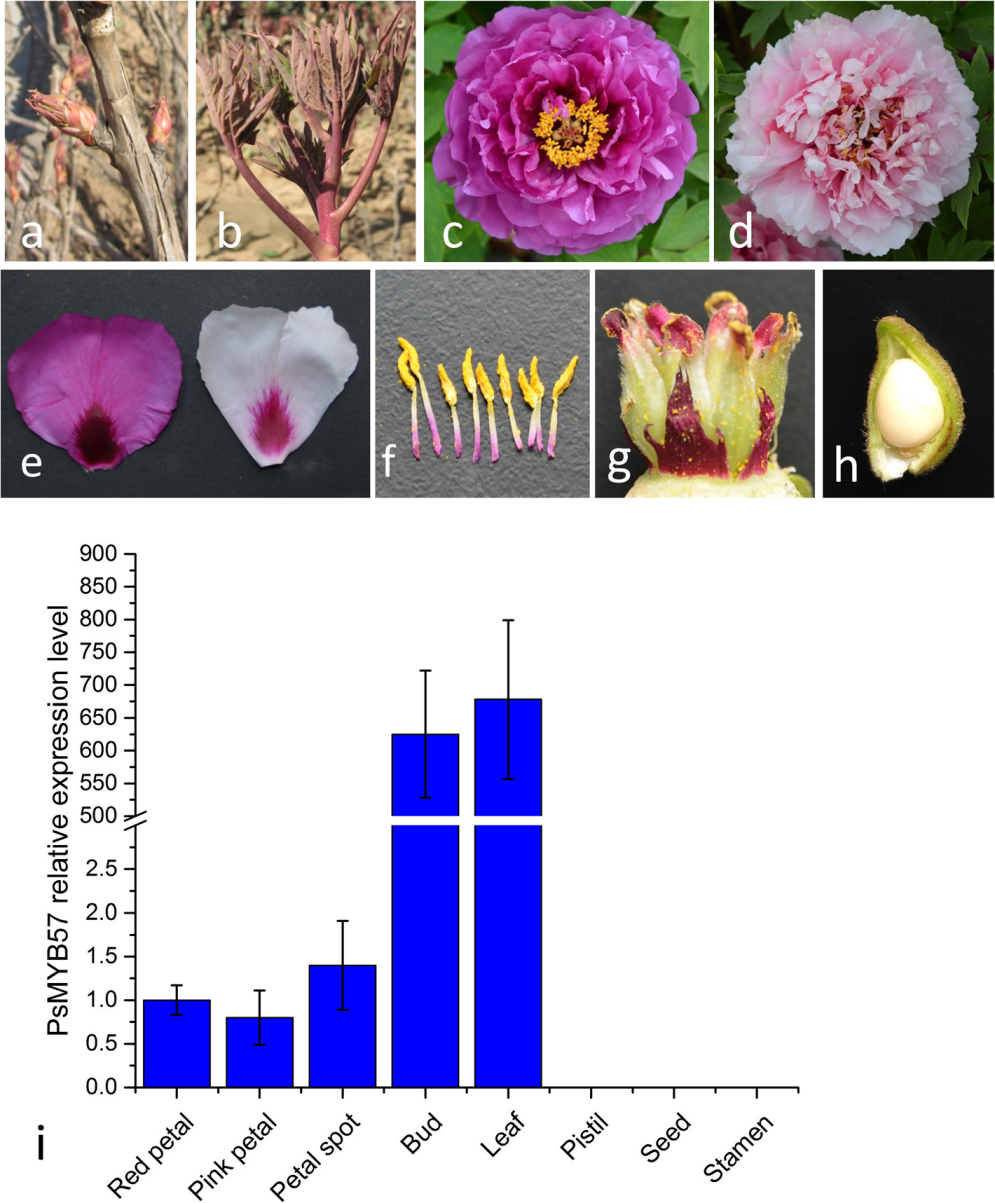
Tissues color of tree peony and transcription level analysis of *PsMYB57*. **a** to **h** display colors of bud, young leaf, red petal, pink petal, petal spot, pistil, stamen and seed of tree peony. **i** The expression profile of *PsMYB57* in different tissues of tree peony. *Ubiquitin* was the reference gene to normalize the expression of *PsMYB57*. Each column represents means ±SD from three independent experiments

The alignment of R2R3-MYBs involving in anthocyanins regulation revealed a highly conserved R2R3 repeat domain in the N-terminal of PsMYB57, and a [DE]Lx2[RK]x3Lx6Lx3R motif, which is necessary for interaction with bHLH proteins, was present in the R3 repeat (Fig. S2a). While in the C-terminal variable region, we found a signature motif KPXPR(S/T) F, which is specific to MYB proteins that activate anthocyanin biosynthesis. The phylogenetic analysis of R2R3-MYBs from different plant species showed that PsMYB57 was clustered into the clade of anthocyanin, and was more closely to Ruby, a MYB transcription factor involved in the anthocyanin pathway in *Citrus sinensis* (Fig. S2b). The R2R3 domain and completed protein sequence of PsMYB57 shared 88 and 51% identity with Ruby, respectively. The sequence analysis indicated that *PsMYB57* was potentially involved in the regulation of the anthocyanin synthetic pathway as well, which was consistent with the analysis of *MYB* gene family in tree peony. In ‘Er Qiao’, pigment was mainly accumulated in tissues of bud, young leaf, red petal and petal spot (Fig. 3a-h). As shown in Fig. 3i, *PsMYB57* was mainly expressed in bud and young leaf, but its transcript was not detectable or expressed at a very low level in other tissues.

### Overexpression of *PsMYB57* induces anthocyanin accumulation in tobacco

**Fig. 4:**
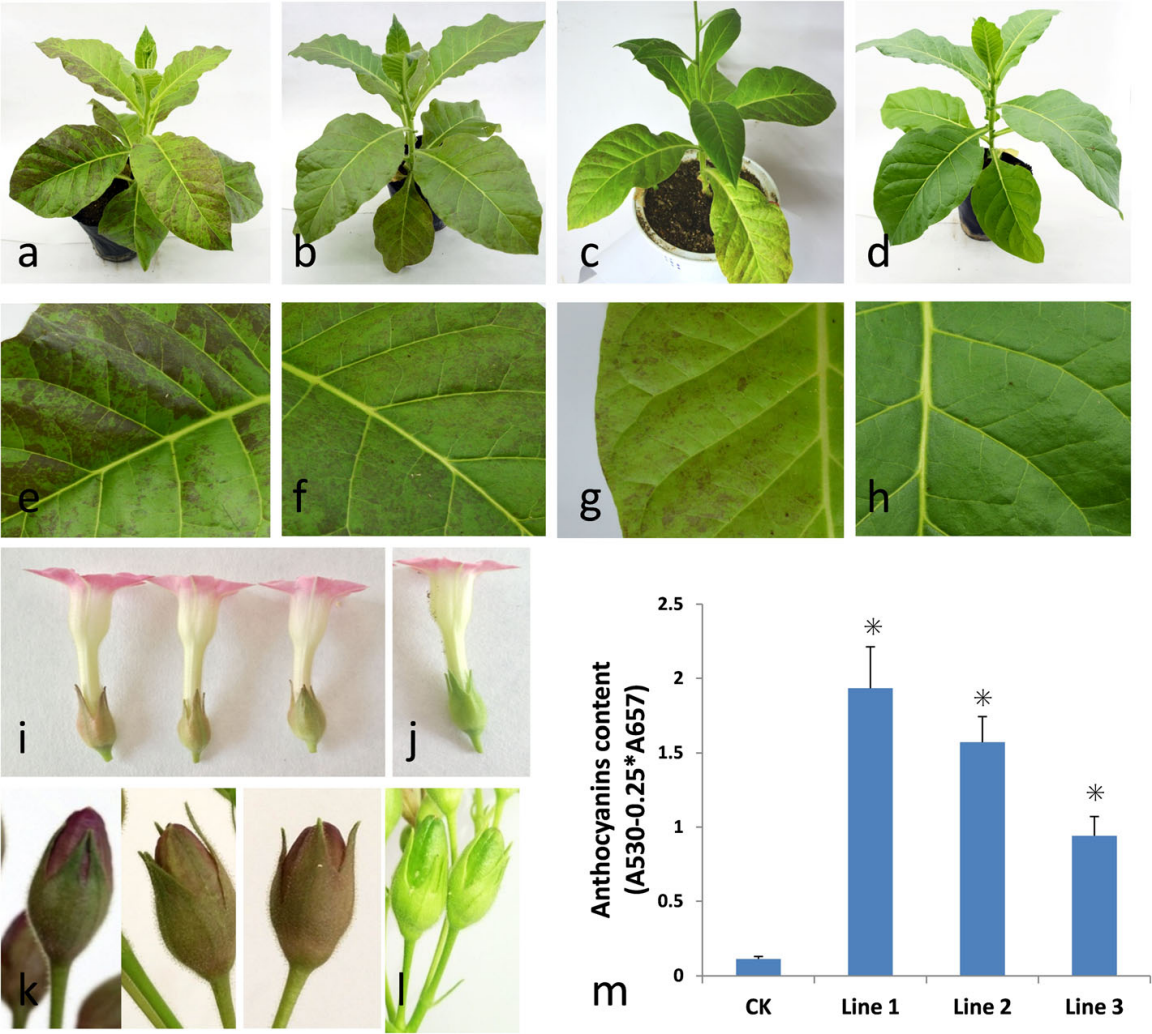
Phenotypic observation and anthocyanin content in *PsMYB57* transgenic tobacco lines. **a** to **c** are the *PsMYB57* transgenic tobacco lines; **d** is the wild-type tobacco line; **e** to **g** are the leaves of the transgenic tobacco lines, **h** is leaf of wild-type tobacco line; **i** is three transgenic flowers; **j** is the wild-type flower; **k** is the sepals of the transgenic lines; **l** is the sepals of the wild-type lines; and **m** is the total anthocyanin content of the leaves in the wild-type and three transgenic lines. Three biological replicates were performed for anthocyanins detection and the asterisk indicates statistical significance (∗*P* < 0.05)

To verify the function of putative anthocyanin regulation genes, coding region sequence of *PsMYB57* was transferred into tobacco under the control of the cauliflower mosaic virus 35S promoter. In the process of obtaining hygromycin-resistant callus, no colored callus was found. The transgenic lines usually display red patches on the leaves (Fig. 4 a–h); we found that three lines displayed significant red patches on the leaves, while eight lines displayed only light red patches. None of the 11 lines had red pigments in the young leaves. In the reproductive organs, the transgenic lines showed red coloring in the sepals and pericarps (Fig. 4 i–l), but no obvious change in flower color was observed. Anthocyanin content was determined in the different plants with dark red or light red patches, and the transgenic lines contained higher levels of anthocyanins, whereas anthocyanins were not detected in the control plants (Fig. 4 m).

### *PsMYB57* promotes the expression of anthocyanin pathway genes in tobacco

**Fig. 5:**
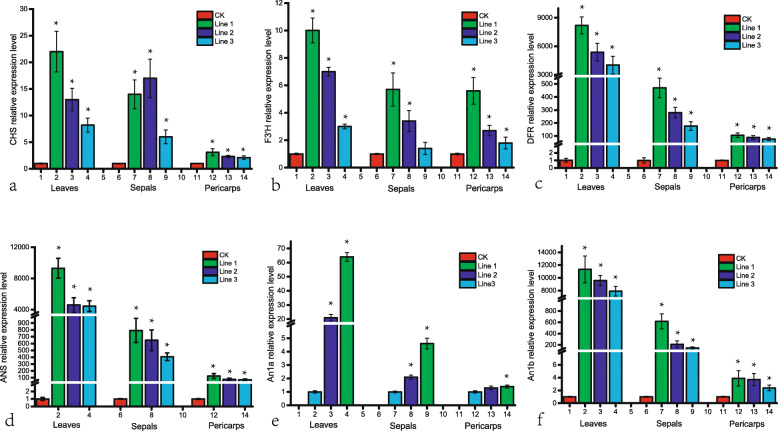
Transcript levels of structural anthocyanin genes and endogenous bHLH genes in wild-type and transgenic tobacco. **a** to **f** are transcription levels of *NtCHS*, *NtF3’H*, *NtDFR* and *NtANS, NtAn1a* and *NtAn1b* in leaves, sepals and pericarps of wild-type and transgenic tobacco lines. Transcription level of *NtAn1a* was not detectable in wild-type lines, and transgenic line 3 used as control in (**e**). CK was wild-type tobacco lines, Line 1 to Line 3 were three *PsMYB57* transgenic tobacco lines. Three T1 generation lines from each transgenic tobacco were collected as samples. *TubulinA1* was used as an internal control to normalize the relative expression levels of the analyzed genes. qRT-PCR analysis was performed with three biological and technical replicates per experiment. An asterisk indicates statistical significance (∗*P* < 0.05)

Quantitative real-time PCR (qRT-PCR) analysis verified that *PsMYB57* was transcribed in the transgenic lines but was not detected in the control plants. In T1 generation of the three *PsMYB57* transgenic tobacco lines, genes transcript levels was detected in tissues of leaves, sepals and pericarps (Fig. 5). Results showed that the transcript levels of one EBG gene (*NtCHS*) and two LBG genes (*NtDFR* and *NtANS*) in the anthocyanin pathway were remarkably increased. *NtF3’H* was highly expressed in detected tissues of *PsMYB57* transgenic lines, but showed only a slight increase in sepals of transgenic line 3. We also detected the transcript levels of two endogenous *bHLH* genes, *NtAn1a* and *NtAn1b*, which facilitate the regulation of anthocyanin biosynthesis by *MYB* in tobacco [36]. The transcription level of *NtAn1a* was not detectable in tissues of wild-type plant. The two *bHLHs* were upregulated in the tissues of all three detected transgenic lines. Overall, structural anthocyanin genes and *bHLH* genes showed similar trends as *PsMYB57* in the transgenic lines.

## Discussion

With the rapid development of high-throughput sequencing technology, whole-genome sequences of more animals and plants have become available. However, the genomes of the majority of species have not yet been sequenced. Furthermore, the genomes of some animals and plants are large and complex and thus pose significant challenges to the sequencing work. To promote functional gene research in species without reference genomes, it is necessary to establish as complete a gene reference database as possible. For instance, in Chinese giant salamander [37], whitefly [38], and *Spodoptera frugiperda* [39], the transcriptomes of different tissues were used to construct a reference gene set. Tree peony has a large genome of about 13 Gb, but complete genome information has not yet been available. Furthermore, *MYB* genes in tree peony are highly limited in the Genbank database. To identify as many *MYB* genes as possible, eight different tree peony tissues were collected and their transcriptomes sequenced to construct a reference gene library, which included 92,837 assemble unigenes. Our data not only provide comprehensive gene sequences for identifying as many *MYB* genes as possible, but also offer a valuable resource to future biological research in tree peony.

Based on the reference transcriptome, a total of 57 *MYBs* were identified in tree peony. Our observed *MYBs* were lower than those reported in plants with reference genomes, as only *MYBs* with complete ORFs were analyzed for subsequent research on gene function. Furthermore, some members with low transcription levels or with spatiotemporal-specific transcription may not be recovered. We found a series of evenly distributed and highly conserved Trp residues in our 57 MYB members, which have been reported in pineapple [11], watermelon [40], and sweet orange [6]. A conserved Trp residue is regarded as a landmark of the MYB domain and plays key roles in sequence-specific DNA binding [5, 41]. In addition to the conserved Trp residues, there are nine amino acids that were completely conserved in different positions. We suggest that the MYB domain in tree peony is highly evolutionarily conserved.

The functions of many R2R3 MYBs have been characterized and several members are associated with the control of plant-specific processes, including primary and secondary metabolism, developmental processes, cell fate and identity, and stress responses [9]. Based on the phylogenetic analysis, the 57 MYBs in tree peony and 124 MYBs in *Arabidopsis* were classified into 35 clades. In general, members within the same subfamily are assumed to have recent common evolutionary origins and usually display similar sequences and functions [42]. Our results provide a reference for exploring the functions of MYBs. For example, PsMYB57 was grouped together with *Arabidopsis* AtMYB75 and AtMYB90 in clade 1, which is associated with anthocyanin regulation [43]. PsMYB54 and PsMYB55 were grouped into clade 3 with the *Arabidopsis* AtMYB0, AtMYB66, and AtMYB33, which represent the functional clade of trichome initiation [44–46]. PsMYB10, PsMYB50, and PsMYB51 were found in group C29 and are associated with stress responses [47]. Two clades (C6 and C9) do not possess *Arabidopsis* MYB members, suggesting that these proteins might have specialized roles that were either lost in *Arabidopsis* or gained after divergence from the last common ancestor.

Flower color is an important economic trait that attracts many breeders in tree peony. In numerous plants, *MYB* genes have been verified to regulate the flavonoid pathway and are responsible for tissue color. The heterologous expression of *MYB* genes in tobacco drastically elevates anthocyanin contents in the leaves, as does *VlMYBA2* in grape [48] and *MaAN2* in grape hyacinth [18]. Additionally, the stable tobacco transformants of *MdMYBA*, which control anthocyanin biosynthesis in apple, do not accumulate pigments in the leaves [49]. MYB usually interacts with a bHLH protein to form a complex to regulate anthocyanin structural genes, and a previous study [50] suggested that the absence of anthocyanins in *MdMYBA* transgenic lines is due to a lack of a *bHLH* partner. In our study, the overexpression of *PsMYB57* upregulated structural anthocyanin genes and two endogenous *bHLH* genes, *NtAn1a* and *NtAn1b*, in tobacco. We suggested that *NtAn1a* and *NtAn1b* are the partners of *PsMYB57* in the regulation of structural genes. We note that the leaf color of the *PsMYB57* transgenic lines differed from the reported lines transformed with *VlMYBA2* and *MaAN2*, in which the full leaves were dark red in color. One possible explanation is that the PsMYB57-NtbHLH complex may be unstable or have lower activity, and a tree peony *bHLH* partner is necessary for the full functioning of *PsMYB57* in tobacco. Overall, the overexpression of *PsMYB57* upregulated structural anthocyanin genes in tobacco, leading to anthocyanin accumulation. The results indicated that *PsMYB57* is an important anthocyanin regulator in tree peony.

In the tree peony variety ‘Er Qiao’, the bud, young leaf, and flower tissues typically display a red pigment, and the petal spot is a dark red color. However, *PsMYB57* was mainly expressed in the bud and young leaf. We suggested that *PsMYB57* mainly regulates color in the bud and young leaves, and thus unknown *MYB* genes might exist to take charge of flower color.

## Conclusions

We constructed a comprehensive reference transcriptome of tree peony, including 92,837 unigenes, and identified 57 *R2R3-MYB* genes containing full-length ORFs. Functional prediction of the *R2R3 MYBs* verified that *PsMYB57* regulates anthocyanin biosynthesis in the buds and young leaves in tree peony. Our study provides a basis for the exploitation of candidate *MYB* genes for the genetic engineering of tree peony. Furthermore, *PsMYB57* can contribute to further researches on the improvement of flower colorization.

## Supplementary Information

**Additional file 1:** **Table S1.** Primers used for qRT-PCR analysis of tree peony and transgenic tobacco samples.

**Additional file 2:** **Table S2.** Primers used for PCR amplification of MYB genes.

**Additional file 3:** **Table S3.** Clean reads quality metrics of sequencing project.

**Additional file 4:** **Table S4.** Unigene annotation in public databases.

**Additional file 5:** **Table S5.** MYB deduced amino acid sequence characteristics and predicted subcellular location of genes.

**Additional file 6:** **Figure S1.** The top sequence matches based on the Nr annotation.

**Additional file 7:** **Figure S2.** Sequence alignment and phylogenetic analysis of PsMYB57 and its orthologs. (a) Alignment of the deduced amino acid sequences of PsMYB57 and other R2R3 MYBs associated with anthocyanin from different plant species. The R2 and R3 domain are indicated above the alignment. The motif [DE]Lx2[RK]x3Lx6Lx3R in the R3 repeat is indicated by dark triangles. The motif KPXPR(S/T) F is shown by a red box. (b) Phylogenetic tree was built using the neighbor-joining method using MEGA 5 software, and bootstrap value was set to 1000. PsMYB57 was marked by a red box. Putative functions of all R2R3 MYBs are listed on the right.

## Data Availability

The datasets supporting the conclusions of this article are available in the Genome Sequence Archive in BIG Data Center, Beijing Institute of Genomics (BIG), Chinese Academy of Sciences, with the accession numbers CRA001327 (http://bigd.big.ac.cn/gsa.), and the GenBank data libraries with the accession numbers MK377190-MK377244.

## Abbreviations

MYB: Myeloblastosis
TFs: Transcription factors;
qRT-PCR: Quantitative real-time PCR.
DBD: DNA binding domain
HTH: Helix-turn-helix
Trp: Tryptophans
EBG: Early biosynthetic gene
LBG: Late biosynthetic gene
TADs: Transacting domains
GO: Gene Ontology
KEGG: Kyoto Encyclopedia of Genes and Genomes
KOG: Eukaryotic orthologous group
PAs: Proanthocyanidins
