# Supplementary material for: Functional identification of PsMYB57 involved in anthocyanin regulation of tree peony
Source: BMC Genet. 2020 Nov 16;21:124. doi: 10.1186/s12863-020-00930-7 (PMC7667756; doi:10.1186/s12863-020-00930-7)
Supplement: Supplementary file 1 — Additional file 1: Table S1. Primers used for qRT-PCR analysis of tree peony and transgenic tobacco samples. [file 12863_2020_930_MOESM1_ESM.docx]

Table S1 Primers used for qRT-PCR analysis of tree peony and transgenic tobacco samples

| Primer name | Primer sequence(5'-3') |
| --- | --- |
| PsMYB57-F | TGGCGAAGGGGATCAAGTAG |
| PsMYB57-R | TTTGTCCTGTCTCTGGTGCA |
| PsUBI-F | GACCTATACCAAGCCGAAG |
| PsUBI-R | CGTTCCAGCACCACAATC |
| NtCHS-F | TGACACCCACTTGGATAGTTTAG |
| NtCHS-R | CGACCTCTGGAATTGGATCAG |
| NtF3’H-F | AGGCTCAACACTTCTCGT |
| NtF3’H-R | CATCAACTTTGGGCTTCT |
| NtDFR-F | AACCAACAGTCAGGGGAATG |
| NtDFR-R | TTGGACATCGACAGTTCCAG |
| NtANS-F | TGGCGTTGAAGCTCATACTG |
| NtANS-R | GGAATTAGGCACACACTTTGC |
| NtAn1a-F | ACCATTCTCGAACACCGAAG |
| NtAn1a-R | TGCTAGGGCACAATGTGAAG |
| NtAn1b-F | CTTGAACACTTCTCAAACCGA |
| NtAn1b-R | TGCTAGGGCACAATGTGAAG |
| NtTubA1-F | CTCCTATGCTCCTGTCATTTC |
| NtTubA1-R | GGCGAGGATCACACTTAAC |
