# Supplementary material for: Functional identification of PsMYB57 involved in anthocyanin regulation of tree peony
Source: BMC Genet. 2020 Nov 16;21:124. doi: 10.1186/s12863-020-00930-7 (PMC7667756; doi:10.1186/s12863-020-00930-7)
Supplement: Supplementary file 2 — Additional file 2: Table S2. Primers used for PCR amplification of MYB genes. [file 12863_2020_930_MOESM2_ESM.docx]

Table S2 Primers used for PCR amplification of MYB genes

| Gene | Forward primer | Reverse primer |
| --- | --- | --- |
| PsMYB4 | GAGATGAAAGATATAGGAGTC | GACGGTACCTTTATGTTTCTC |
| PsMYB10 | TCTGTAAATGGAAGCAATGAA | CCTAAATCTGAAAAAGACCAA |
| PsMYB14 | AGTGAGGAGTGATGAGAAACC | GAACCGTCTTGACAGGTAATT |
| PsMYB21 | CTGATTGATTCTTTTGTCCAA | CCTCACCTTCAAAATCTCTAA |
| PsMYB27 | TCTAGGGATCAACGTTAAGAA | AGGTTGAAACTTGAACTCCTT |
| PsMYB30 | TTGTTGATTTTTCACTCCAGA | GCTATTAACATTTTCTCCAGT |
| PsMYB35 | GTATTATCTCTGATTCTAGTA | CTGATCATTAACTAACATGAA |
| PsMYB43 | ATCTCCTCATCTTTTATTGTA | ACATTTAGTTTTTCACACAAC |
| PsMYB49 | TGTTAGAGAGATTGGTATCCA | ATAGGATCCATCTTGGAGTCA |
| PsMYB54 | AAGGAAACAAAATAAGAGCAA | GTGATTATTTACAAACCATGA |
