# Supplementary material for: Functional identification of PsMYB57 involved in anthocyanin regulation of tree peony
Source: BMC Genet. 2020 Nov 16;21:124. doi: 10.1186/s12863-020-00930-7 (PMC7667756; doi:10.1186/s12863-020-00930-7)
Supplement: Supplementary file 3 — Additional file 3: Table S3. Clean reads quality metrics of sequencing project. [file 12863_2020_930_MOESM3_ESM.docx]

Table S3 Clean reads quality metrics of sequencing project

| Sample | Total clean reads (Mb) | Total clean bases (Gb) | Clean reads Q20 (%) | Clean Reads Q30(%) |
| --- | --- | --- | --- | --- |
| Leaf | 67.15 | 6.72 | 96.92 | 88.59 |
| Pink petal | 65.92 | 6.59 | 96.83 | 88.59 |
| Pistil | 66.75 | 6.68 | 97.00 | 88.86 |
| Red petal | 66.44 | 6.64 | 97.06 | 89.22 |
| Seed | 65.21 | 6.52 | 96.62 | 87.98 |
| Shoot | 65.31 | 6.53 | 97.28 | 89.59 |
| Spot | 65.98 | 6.60 | 96.95 | 88.82 |
| Stamen | 65.41 | 6.54 | 96.71 | 88.16 |
