# Supplementary material for: Functional identification of PsMYB57 involved in anthocyanin regulation of tree peony
Source: BMC Genet. 2020 Nov 16;21:124. doi: 10.1186/s12863-020-00930-7 (PMC7667756; doi:10.1186/s12863-020-00930-7)
Supplement: Supplementary file 4 — Additional file 4: Table S4. Unigene annotation in public databases. [file 12863_2020_930_MOESM4_ESM.docx]

Table S4 Unigene annotation in public databases

| Database | Number | Percentage |
| --- | --- | --- |
| Nr | 45,411 | 48.91% |
| Nt | 33,144 | 35.70% |
| Swissprot | 30,487 | 32.84% |
| KEGG | 34,212 | 36.85% |
| KOG | 35,469 | 38.21% |
| Interpro | 37,021 | 39.88% |
| GO | 23,355 | 25.16% |
| Total | 92,837 | 100% |
