# Supplementary material for: Functional identification of PsMYB57 involved in anthocyanin regulation of tree peony
Source: BMC Genet. 2020 Nov 16;21:124. doi: 10.1186/s12863-020-00930-7 (PMC7667756; doi:10.1186/s12863-020-00930-7)
Supplement: Supplementary file 5 — Additional file 5: Table S5. MYB deduced amino acid sequence characteristics and predicted subcellular location of genes. [file 12863_2020_930_MOESM5_ESM.doc]

Table S5 MYB deduced amino acid sequence characteristics and predicted subcellular location of genes

| Gene name | Number of amino acids | Molecular weight | Theoretical pI | Subcellular localization |
| --- | --- | --- | --- | --- |
|  |  |  |  |  |
| PsMYB1 | 344 | 38036.70 | 5.92 | Nucleus. |
| PsMYB2 | 305 | 34098.07 | 7.70 | Nucleus. |
| PsMYB3 | 191 | 21940.11 | 10.22 | Nucleus. |
| PsMYB4 | 418 | 45195.41 | 5.22 | Nucleus. |
| PsMYB5 | 500 | 54179.59 | 5.63 | Nucleus. |
| PsMYB6 | 225 | 25705.17 | 9.26 | Nucleus. |
| PsMYB7 | 480 | 52437.75 | 6.43 | Nucleus. |
| PsMYB8 | 327 | 37175.28 | 5.78 | Nucleus. |
| PsMYB9 | 560 | 61363.04 | 5.24 | Nucleus. |
| PsMYB10 | 230 | 25628.6 | 9.67 | Nucleus. |
| PsMYB11 | 465 | 51773.3 | 5.06 | Nucleus. |
| PsMYB12 | 493 | 53321.87 | 5.8 | Nucleus. |
| PsMYB13 | 289 | 32809.28 | 6.31 | Nucleus. |
| PsMYB14 | 305 | 34096.03 | 7.7 | Nucleus. |
| PsMYB15 | 199 | 22808.78 | 8.53 | Nucleus. |
| PsMYB16 | 200 | 22696.65 | 8.64 | Nucleus. |
| PsMYB17 | 177 | 20672.10 | 9.76 | Nucleus. |
| PsMYB18 | 483 | 54357.28 | 6.19 | Nucleus. |
| PsMYB19 | 286 | 32503.61 | 6.26 | Nucleus. |
| PsMYB20 | 293 | 31856.53 | 5.83 | Nucleus. |
| PsMYB21 | 370 | 41881.43 | 7.99 | Nucleus. |
| PsMYB22 | 278 | 32020.38 | 9.28 | Nucleus. |
| PsMYB23 | 278 | 31650.42 | 5.52 | Nucleus. |
| PsMYB24 | 315 | 35490.50 | 7.17 | Nucleus. |
| PsMYB25 | 297 | 33881.16 | 5.18 | Nucleus. |
| PsMYB26 | 302 | 33870.04 | 6.83 | Nucleus. |
| PsMYB27 | 327 | 36014.28 | 6.11 | Nucleus. |
| PsMYB28 | 226 | 25787.36 | 8.73 | Nucleus. |
| PsMYB29 | 278 | 31056.08 | 8.53 | Nucleus. |
| PsMYB30 | 408 | 44414.34 | 5.72 | Nucleus. |
| PsMYB31 | 333 | 37289.49 | 5.96 | Nucleus. |
| PsMYB32 | 260 | 29413.92 | 5.69 | Nucleus. |
| PsMYB33 | 170 | 19330.29 | 9.58 | Nucleus. |
| PsMYB34 | 308 | 34232.69 | 9.11 | Nucleus. |
| PsMYB35 | 291 | 32976.11 | 6.01 | Nucleus. |
| PsMYB36 | 255 | 28318.18 | 8.64 | Nucleus. |
| PsMYB37 | 279 | 31440.84 | 5.47 | Nucleus. |
| PsMYB38 | 373 | 42323.18 | 8.83 | Nucleus. |
| PsMYB39 | 378 | 42734.50 | 4.95 | Nucleus. |
| PsMYB40 | 424 | 47362.98 | 6.83 | Nucleus. |
| PsMYB41 | 339 | 38347.05 | 5.25 | Nucleus. |
| PsMYB42 | 193 | 22294.41 | 9.30 | Nucleus. |
| PsMYB43 | 379 | 42645.65 | 4.86 | Nucleus. |
| PsMYB44 | 386 | 43571.22 | 8.01 | Nucleus. |
| PsMYB45 | 315 | 35317.17 | 6.56 | Nucleus. |
| PsMYB46 | 305 | 34749.12 | 5.18 | Nucleus. |
| PsMYB47 | 218 | 24817.14 | 9.23 | Nucleus. |
| PsMYB48 | 215 | 24450.61 | 8.97 | Nucleus. |
| PsMYB49 | 230 | 25928.34 | 8.83 | Nucleus. |
| PsMYB50 | 278 | 30622.55 | 8.46 | Nucleus. |
| PsMYB51 | 325 | 35424.77 | 8.66 | Nucleus. |
| PsMYB52 | 210 | 24757.43 | 9.46 | Nucleus. |
| PsMYB53 | 268 | 30897.41 | 5.65 | Nucleus. |
| PsMYB54 | 227 | 25390.55 | 6.53 | Nucleus. |
| PsMYB55 | 210 | 24139.35 | 6.98 | Nucleus. |
| PsMYB56 | 354 | 40823.53 | 9.47 | Nucleus. |
| PsMYB57 | 269 | 30334.58 | 9.19 | Nucleus. |
