# Supplementary figures and images for: Functional identification of PsMYB57 involved in anthocyanin regulation of tree peony
Source: BMC Genet. 2020 Nov 16;21:124. doi: 10.1186/s12863-020-00930-7 (PMC7667756; doi:10.1186/s12863-020-00930-7)

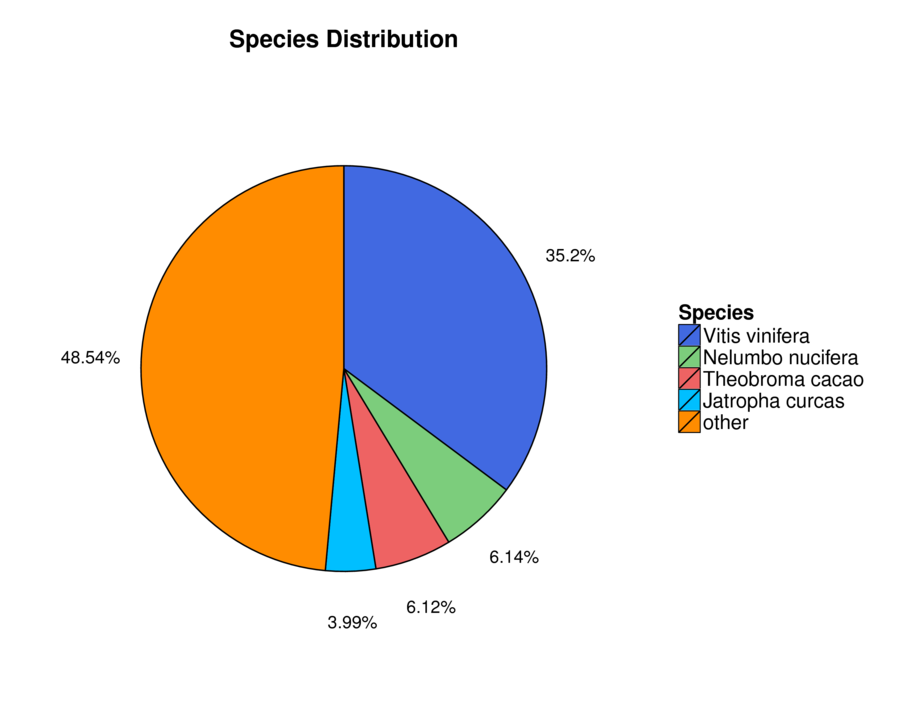

Supplement: Supplementary file 6 — Additional file 6: Figure S1. The top sequence matches based on the Nr annotation. [file 12863_2020_930_MOESM6_ESM.png]

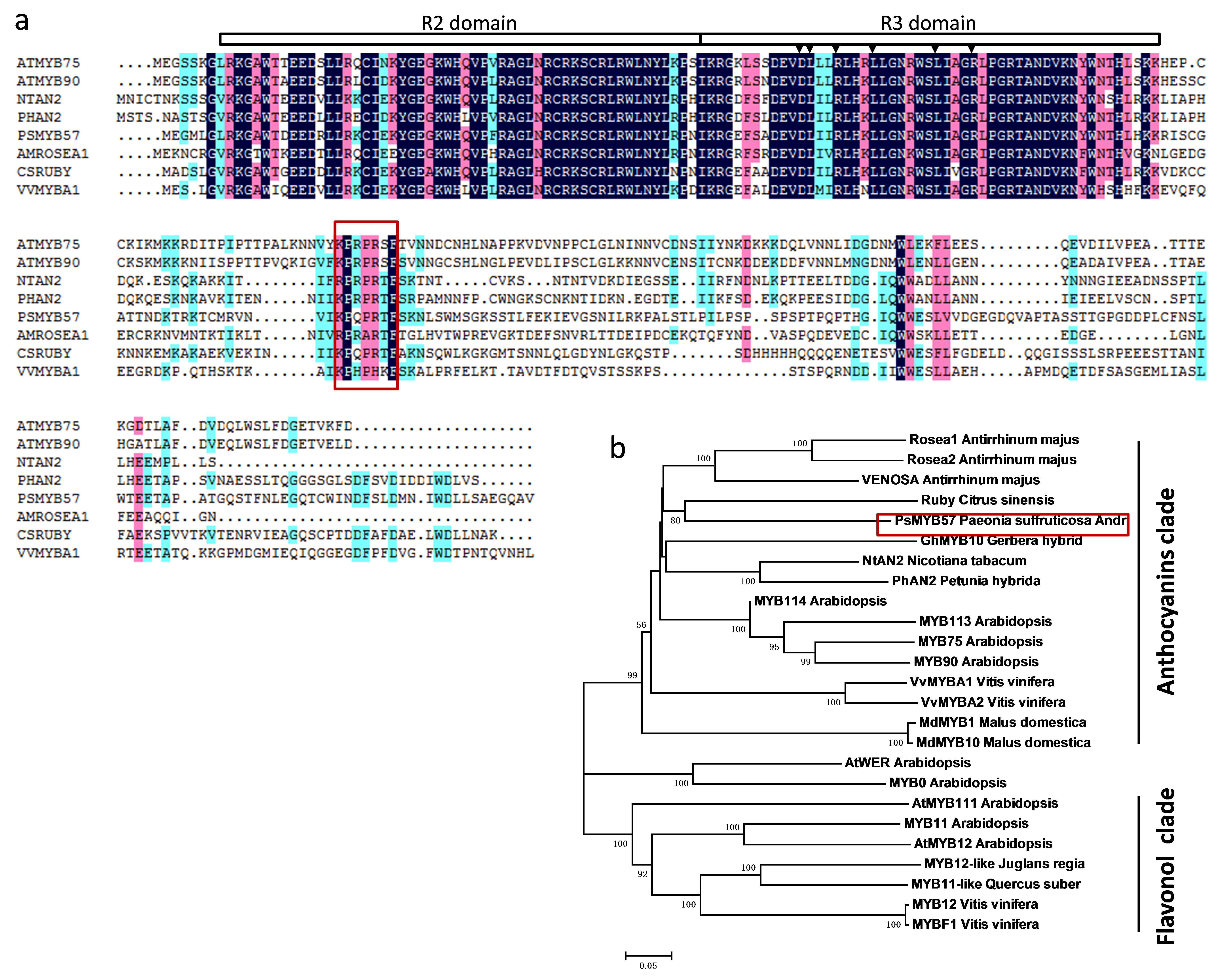

Supplement: Supplementary file 7 — Additional file 7: Figure S2. Sequence alignment and phylogenetic analysis of PsMYB57 and its orthologs. (a) Alignment of the deduced amino acid sequences of PsMYB57 and other R2R3 MYBs associated with anthocyanin from different plant species. The R2 and R3 domain are indicated above the alignment. The motif [DE]Lx2[RK]x3Lx6Lx3R in the R3 repeat is indicated by dark triangles. The motif KPXPR(S/T) F is shown by a red box. (b) Phylogenetic tree was built using the neighbor-joining method using MEGA 5 software, and bootstrap value was set to 1000. PsMYB57 was marked by a red box. Putative functions of all R2R3 MYBs are listed on the right. [file 12863_2020_930_MOESM7_ESM.png]
